# Supplementary material for: Arabidopsis S2Lb links AtCOMPASS-like and SDG2 activity in H3K4me3 independently from histone H2B monoubiquitination
Source: Genome Biol. 2019 May 21;20:100. doi: 10.1186/s13059-019-1705-4 (PMC6528313; doi:10.1186/s13059-019-1705-4)
Supplement: Supplementary file 2 — Figure S1. S2La and S2Lb mRNA levels in plant organs. Figure S2. Amino acid sequence alignment of full-length S2La and S2Lb with Swd2 (S. cerevisiae) and Wdr82 (H. sapiens) proteins. Figure S3. Expression of S2La and S2Lb genes in s2la-1 and s2lb-1 mutant plants. Figure S4. Complementation of s2lb-2 morphological phenotypes by pS2Lb::S2Lb-GFP. Figure S5. Decreased H3K4me3 level in S2Lb loss-of-function plants. Figure S6. ChIP-seq quality control and statistical analyses. Figure S7. H3K4me3 over S2Lb-GFP targeted genes. Figure S8. RNA-seq analysis of s2lb-2 mutant seedlings. Figure S9. S2Lb-GFP expression and RNA-seq. Figure S10. S2Lb and SDG2 co-regulate a large set of genes and associate within a high molecular weight complex. Figure S11. Detection of genes losing H3K4me3 enrichment in hub1-3 seedlings targeted by S2Lb-GFP. Figure S12. Genome browser snapshots of H3K4me3 profiles over representative genes. Figure S13. PCR genotyping and ChIP epigenotyping of the hub1-3 plants in the seed batches used for ChIP-seq analyses in this study. (PDF 16889 kb) [file 13059_2019_1705_MOESM2_ESM.pdf]

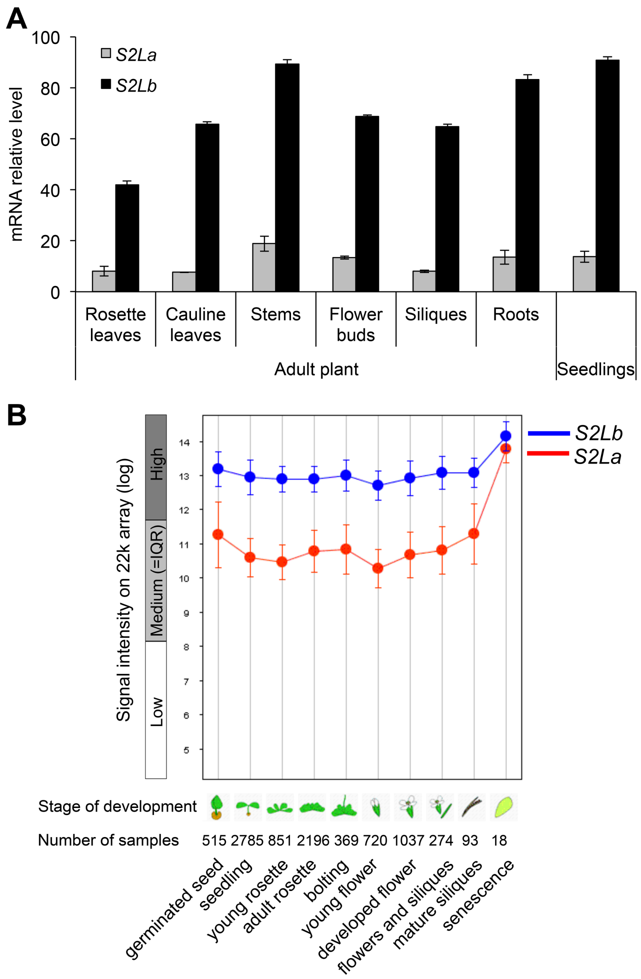

Figure S1. *S2La* and *S2Lb* mRNA levels in plant organs.
A) Relative mRNA levels of *S2La* (grey bars) and *S2Lb* (black bars) in RT-qPCR analyses of different organs from from 10-day-old seedlings or wild-type adult plants (43 DAS). Error bars represent SD from two technical replicates. Transcript levels were normalized to the mean levels of *At2g36060*, *At4g29130* and *At5g13440* housekeeping genes. **B)** Genevestigator (Zimmermann et al., 2004) screenshot showing *S2La* and *S2Lb* gene expression at various developmental stages. Values are given as means +/-SD.

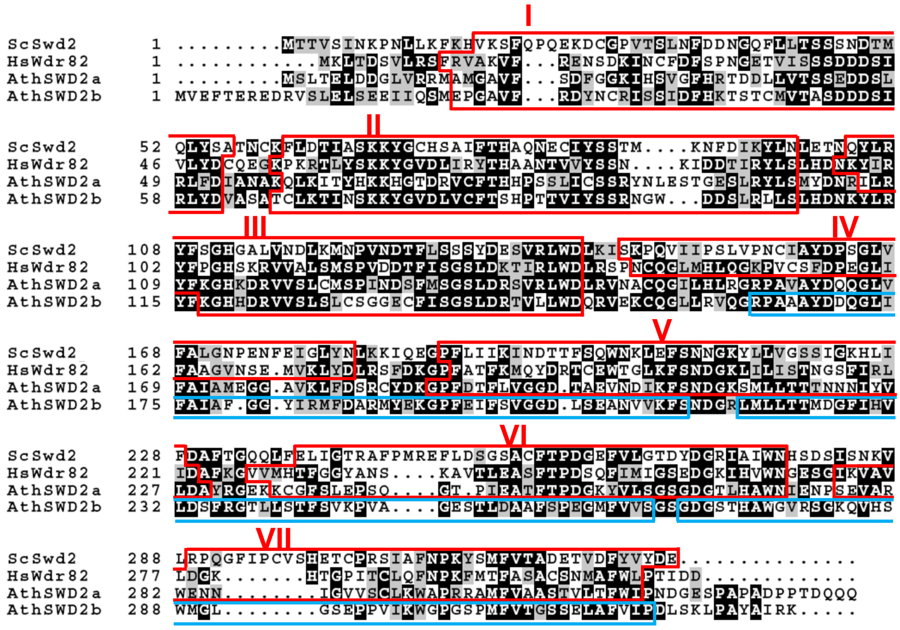

Figure S2. Amino-acids sequence alignment of full-length S2La and S2Lb with Swd2 (*S. cerevisiae*) and Wdr82 (*H. sapiens*) proteins. Numbers refer to amino-acids residues. Identical and similar residues are shaded in black and grey, respectively. WD40 repeats were defined according to WDSP predicting software (Wang et al., 2013). Conserved WD40 repeats are framed in red and numbered from I to VII. Divergent S2Lb WD40 repeats are framed in blue.

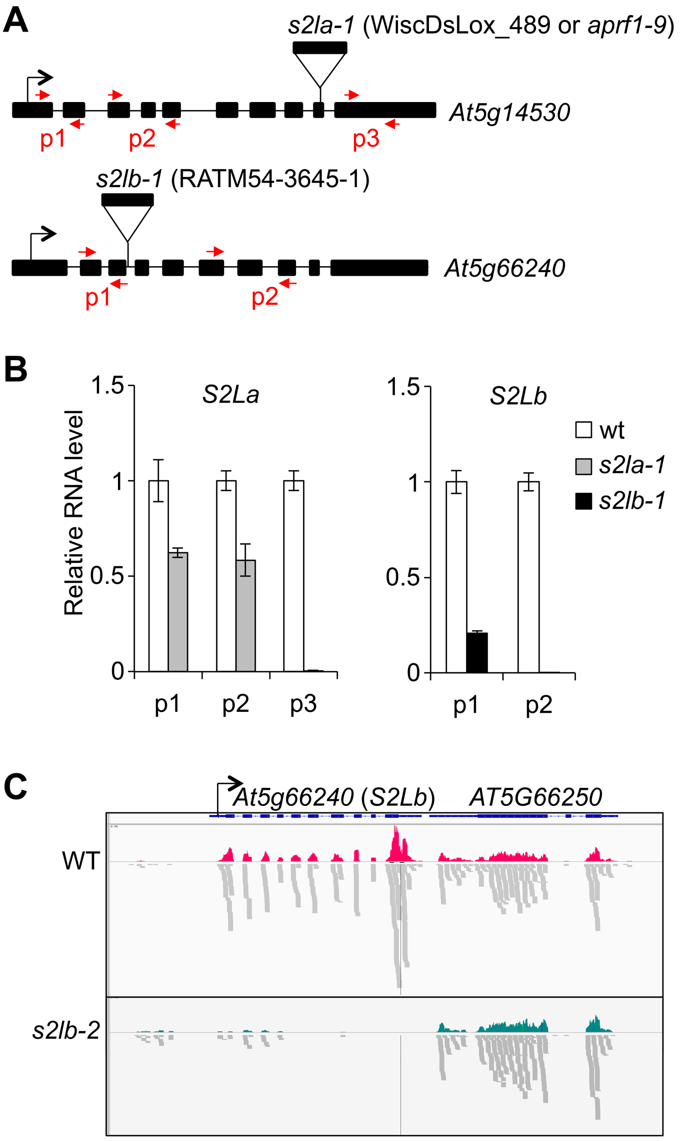

Figure S3. Expression of *S2La* and *S2Lb* genes in *s2la-1* and *s2lb-1* mutant plants.
A. Schematic representation of the *S2La* and the *S2Lb* genes with the localization of the T-DNA insertions in the *s2la-1* and *s2lb-2* lines, respectively. Exons are represented by block boxes. **B.** RT-qPCR analysis of *S2La* and *S2Lb* transcript levels in adult *s2la-1* and *s2lb-1* mutant plants using the primers indicated (p1, p2, p3). RNA levels in the respective wild-type plants are arbitrarily set to 1. Primers positions are indicated in A (red arrows). Error bars represent SD from two technical replicates. Transcript levels were normalized to the mean levels of *At2g36060*, *At4g29130* and *At5g13440* housekeeping genes. **C.** Genome browser snapshot showing mRNA-seq read coverage (upper panels) and read positioning in wild-type and *s2lb-2* seedlings (lower panels).

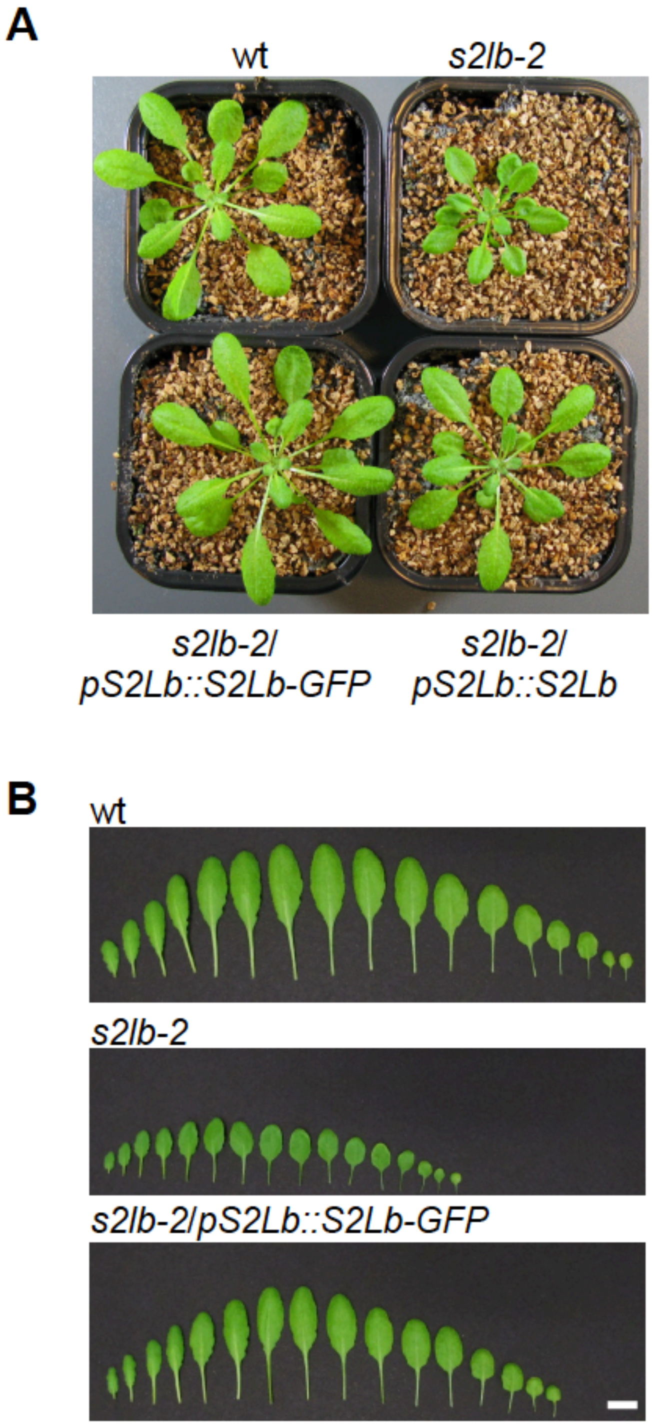

Figure S4. Complementation of *s2lb-2* morphological phenotypes by *pS2Lb::S2Lb* and *pS2Lb::S2Lb-GFP* constructs.
Rosette **A)** and leaf **B)** phenotypes of 4-week-old plants grown under short day conditions. Scale bars, 1 cm.

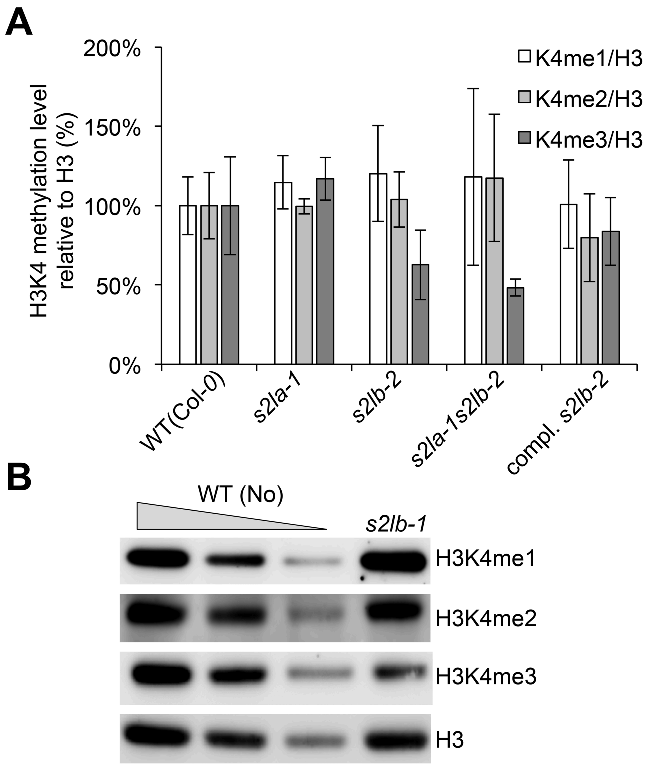

Figure S5. Decreased H3K4me3 level in *S2Lb* loss-of-function plants.
A) Semi-quantitative analysis of H3K4 methylation signals in *S2L* loss-of-function plants. H3K4me1, H3K4me2 and H3K4me3 signal intensity relative to total histone H3 level were quantified using a luminescence imager. Levels in wild-type plants are arbitrarily set to 1. Error bars indicate SD from 3 independent biological replicates. **B.** Same as in (A) using antibodies recognizing H3K4me1, H3K4me2 and H3K4me3 and chromatin extracts from wild-type Nossen and *s2lb-1* plants. Histone H3 is used as loading control for H3K4me3 and H2Bub signals.

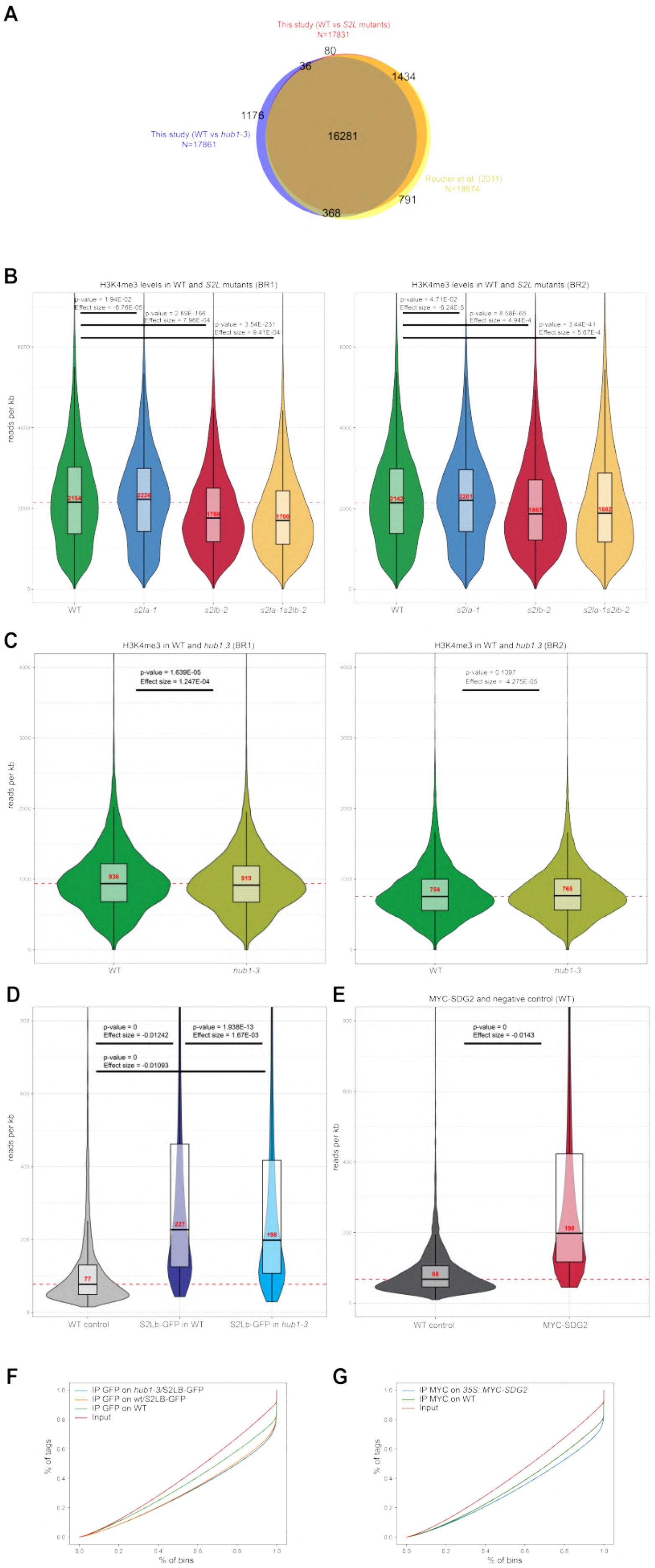

A
This study (WT vs S2L mutants)
N=17831
This study (WT vs hub1-3)
N=17861
Roudier et al. (2011)
N=18874
80
36
1434
1176
16281
791
368
B
H3K4me3 levels in WT and S2L mutants (BR1)
H3K4me3 levels in WT and S2L mutants (BR2)
reads per kb
WT
s2la-1
s2lb-2
s2la-1s2lb-2
C
H3K4me3 in WT and hub1.3 (BR1)
H3K4me3 in WT and hub1.3 (BR2)
p-value = 1.639E-05
Effect size = 1.247E-04
reads per kb
WT
hub1-3
D
p-value = 0
Effect size = -0.01242
p-value = 1.938E-13
Effect size = 1.67E-03
p-value = 0
Effect size = -0.01093
reads per kb
WT control
S2Lb-GFP in WT
S2Lb-GFP in hub1-3
E
MYC-SDG2 and negative control (WT)
p-value = 0
Effect size = -0.0143
reads per kb
WT control
MYC-SDG2
F
IP GFP on hub1-3/S2LB-GFP
IP GFP on wt/S2LB-GFP
IP GFP on WT
Input
% of tags
% of bins
G
IP MYC on 35S::MYC-SDG2
IP MYC on WT
Input
% of tags
% of bins

Figure S6. Validation and statistical analysis of ChIP-seq datasets.

A) H3K4me3-marked genes identified in this study as compared to those in Roudier et *al.* (2011). The Venn diagram shows the overlap between H3K4me3-marked genes in this study (Additional file 3) and in Roudier et al (2011). **B)** Distribution of H3K4me3 short reads in wild-type and *S2L* mutant plants over genes overlapping with ChIP peaks identified in WT for the biological replicate 1 (BR1; left panel) and biological replicate 2 (BR2; right panel). Median read coverage is indicated in red. P-values for pairwise Mann–Whitney U test comparisons with the WT sample are given. The pValues indicate that *s2lb-2* and *s2la-1s2lb-2* mutants display significant lower levels than compared to WT, whereas *s2la-1* mutant plants have retained most of the mark. **C)** Same analysis than B) for *hub1-3* mutant plants. A measure of the effect size is also reported and indicates that the read density over marked genes is not different in mutant compared to wild-type plants. **D)** Distribution of short reads in wild-type plants (WT control), *WT/S2Lb::S2Lb-GFP* and *hub1-3/S2Lb::S2Lb-GFP* lines. For both transgenic lines, the IPs show a robust GFP signal enrichment as compared to the WT negative control, which is slightly weaker in *hub1-3* mutant plants. **E)** Same analysis than D) for MYC-SDG2 enrichment in *35S::MYC-SDG2* plants as compared to wild-type plants (WT control). The IP displays a strong enrichment in the transgenic line as compared to wild-type plants. **F)** Fingerprint analysis of S2Lb-GFP ChIP-seq. Cumulative percentage of tag in IP and Input samples over 500,000 randomly chosen 1000-bp genomic bins computer according to Diaz at al., 2012 (Stat. Appl. Genet. Mol. Biol., doi: 10.1515/1544-6115.1750). Reads are evenly distributed over all bins in Input and wild-type negative control samples, producing a linear cumulative distribution. Conversely, samples from the transgenic lines display a curve cumulative distribution, indicating that the highest-ranking bins (peaks, rightmost part of the curve) contain a larger fraction of short reads compared to the remaining bins (background). **G)** Same analysis than F) for MYC-SDG2 ChIP-seq.

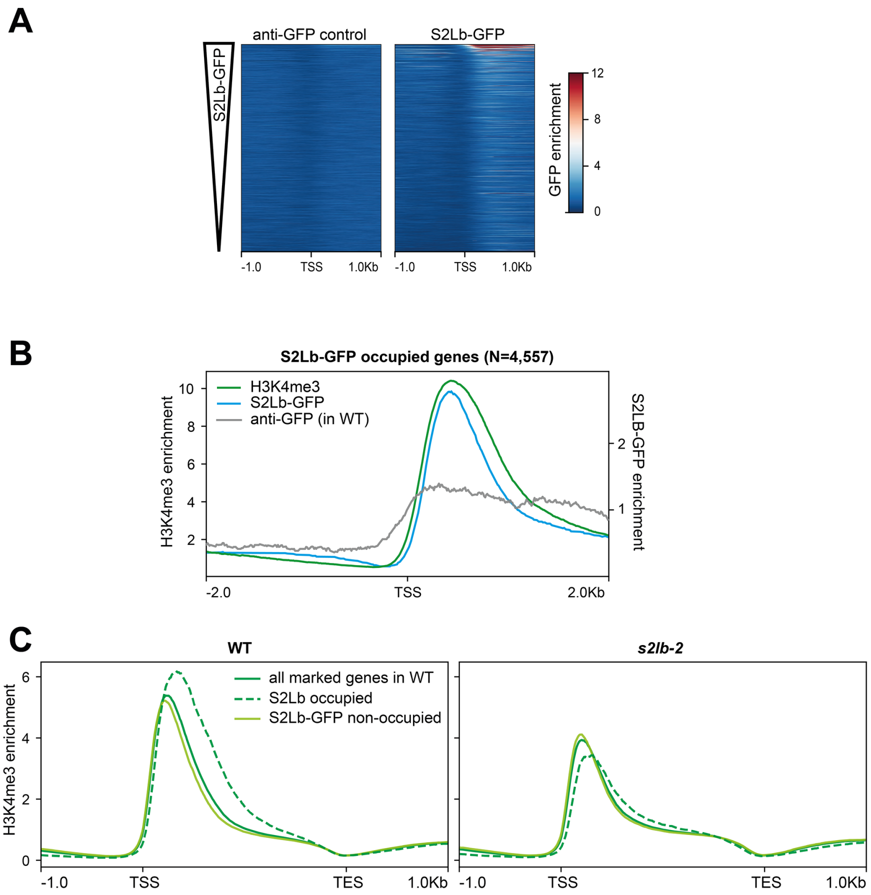

Figure S7. H3K4me3 over S2Lb-GFP targeted genes.
A) S2Lb median enrichment over the genes marked by H3K4me3 in WT plants (N=17,831). Genes are equally ranked from top to bottom in each plant line according to median enrichment of S2Lb-GFP in the right panel. **B)**. Metaplot showing the median enrichment in H3K4me3 in WT plants (green line) or of S2LB-GFP (blue line) over the 4,557 genes targeted by H3K4me3. Background signal is shown by performing an anti-GFP ChIP-seq on wild-type plants grown simultaneously. **C)** Genes targeted by S2LB are globally highly marked by H3K4me3. Metaplots display H3K4me3 median enrichment in wild-type (left panel) or *s2lb-2* (right panel) over all H3K4me3-marked genes in WT seedlings (N=17,831; black line), and the ones occupied by S2Lb-GFP (N=4,557; yellow line) or marked by H3K4me3 but not occupied by S2Lb (N=13,455; blue line).

A **B**

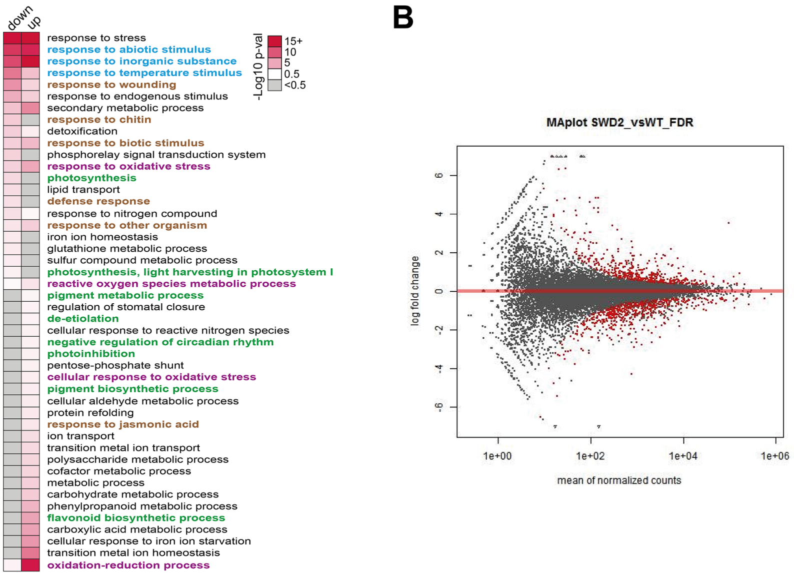

C

	Upregulated genes in *s2lb* (N=270; Log2FC >0 & FDR<0.01)			Downregulated genes in *s2lb* (N=391; Log2FC <0 & FDR<0.01)		
	observed	expected	enrichement	observed	expected	enrichement
**H3K4me3 marked genes in WT & *s2lb*** N=16,627	25	*146*	0.2; p < 4.420e-55	188	*211*	0.9; p < 0.011
**H3K4me3 marked genes in WT only** N=1,204	165	*11*	15.6; p < 2.138e-161	85	*15*	5.6; p < 5.798e-39
**H3K4me3 marked genes in *s2lb* only** N=442	6	*4*	1.5; p < 0.196	1	*6*	0.2; p < 0.023
**Not marked** N=13,073	74	*115*	0.6; p < 1.674e-07	117	*166*	0.7; p < 2.150e-107

Figure S8. RNA-seq analysis of *s2lb-2* mutant seedlings. A) Heatmap showing the Gene Ontology (GO) enrichments (-Log10 p-Val < 0.5 in either up or downregulated genes) for DE genes in *s2lb-2* vs wild-type (FDR<0.01; Additional file 5). Grey color indicates -Log10 p-Val < 0.5. **B)** MA-plot showing the result of the DE-Seq2 analysis of the RNA-seq data comparing the transcriptome of wild-type and *s2lb-2* seedlings. 270 and 391 genes were found to be up and downregulated, respectively in the mutant plants compared to the wild-type (Additional file 5; FDR<0.01; red dots). **C)** Table showing the overlap between DE genes in *s2lb-2* vs WT (FDR<0.01; Additional file 5) and H3K4me3 marking in WT and s2lb. The enrichment p-value was calculated through the nemates.org website. Red color indicates a significant enrichment while green indicates a significant depletion.

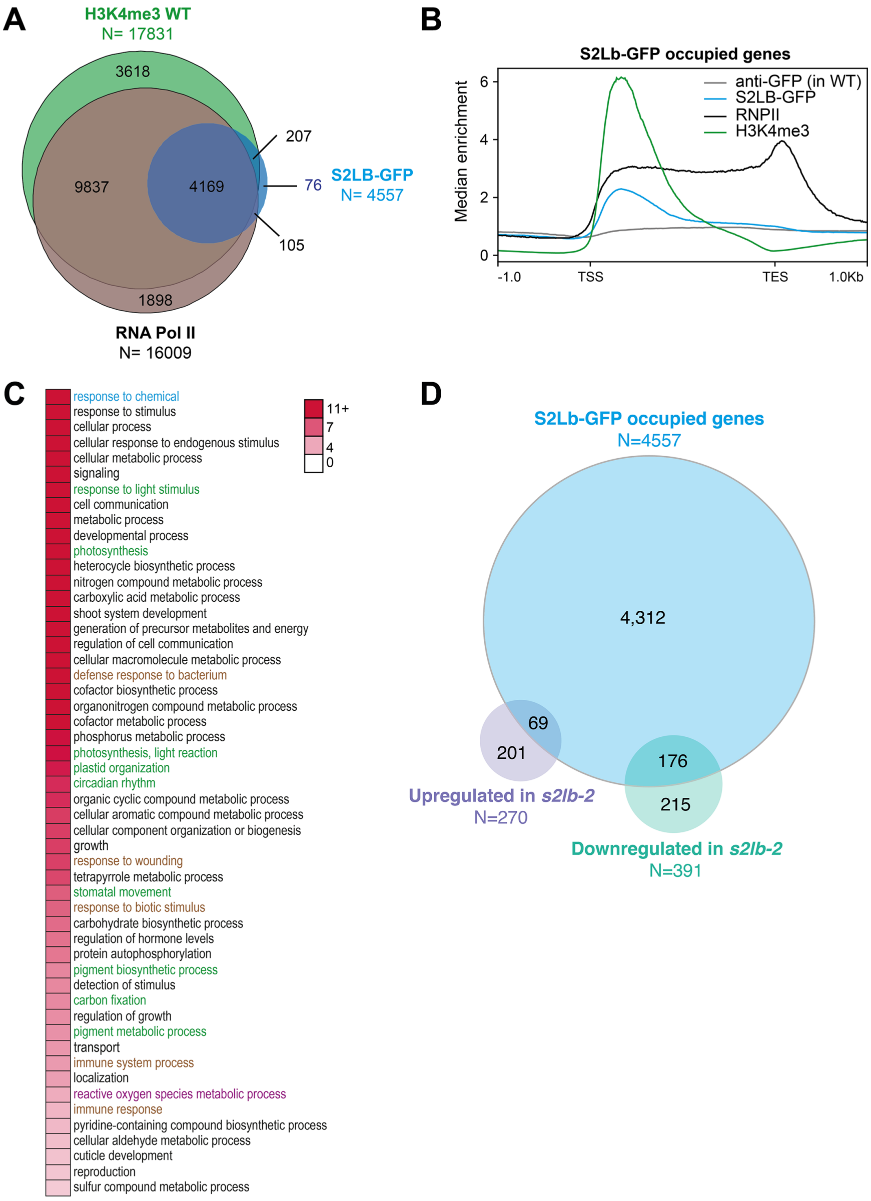

Figure S9. S2Lb-GFP expression and RNA-seq.

A) Overlap between the genes occupied by S2Lb-GFP, H3K4me3 or by RNPII in WT seedlings. Overlap between H3K4me3 and RNPII, representation factor of 1.5 with p-Val<0.000e+00; overlap RNPII and S2Lb-GFP, representation factor of 1.8 with p-Val<0.000e+00; overlap between H3K4me2 and S2Lb-GFP, representation factor of 1.7 with p-Val<0.000e+00. **B)** Median enrichment of H3K4me3, S2Lb-GFP and RNPII over the 4,557 genes occupied by S2Lb-GFP. **C)** Heatmap showing GO terms enrichments (-Log10 p-value) for the 4,557 genes occupied by S2Lb-GFP. The category terms also found enriched for misregulated genes in *s2lb-2* are labeled with by the same color code. **D)** Overlap between the 4,557 genes targeted by S2Lb-GFP and the genes misregulated in *s2lb-2* seedlings (Additional file 5; FDR<0.01). The overlap is significantly enriched (upregulated genes, representation factor of 1.8 with p-Val<1.329e-06; downregulated genes, representation factor of 3.1 with p-Val<3.938e-48).

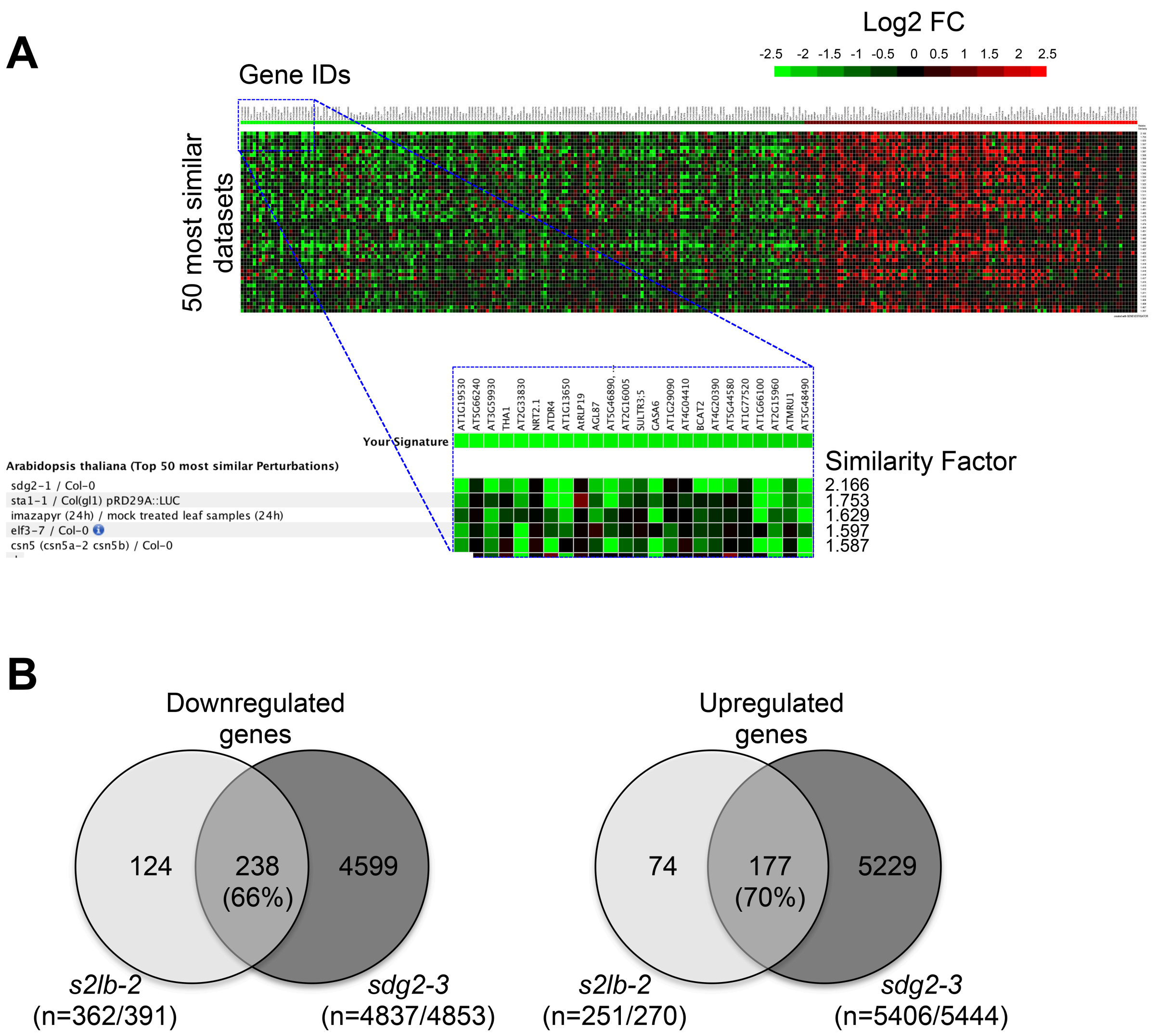

Figure S10. S2Lb and SDG2 co-regulate a large set of genes and associate within a high molecular weight complex.

A) The Genevestigator Signature Tool was used to identify conditions that cause similar gene expression misregulation than the one found in *s2lb-2*. The expression values from genes misregulated in *s2lb-2* mutant seedlings (N=337, LogFC ≥ 1 & FDR ≤ 0.01) were compared to 3,282 other transcriptome experiments (ATH1 arrays) using the Pearson correlation to measure distances. The screenshot shows the 50 most similar datasets (from top to bottom). The inset shows an enlargement over the most downregulated genes in *s2lb-2* seedlings showing that the transcriptome profiling of the *sdg2-1* mutant transcriptome (AT-00435) from Guo et al. (2010) displays the strongest similarity. **B)** Venn-diagrams showing the comparison of down- or upregulated genes in *s2lb-2* (│LogFC│ > 0 and FDR ≤ 0.01) and *sdg2-3* (p-Val < 0.05) mutant seedlings. Only the gene sets for which data are available in different each experiment were kept for further analyses and both numbers are indicated in parentheses. The percentage of genes misregulated both in *s2lb-2* and *sdg2-3* datasets is indicated. The *sdg2-3* transcriptomic data are from (Guo *et al*, 2010).

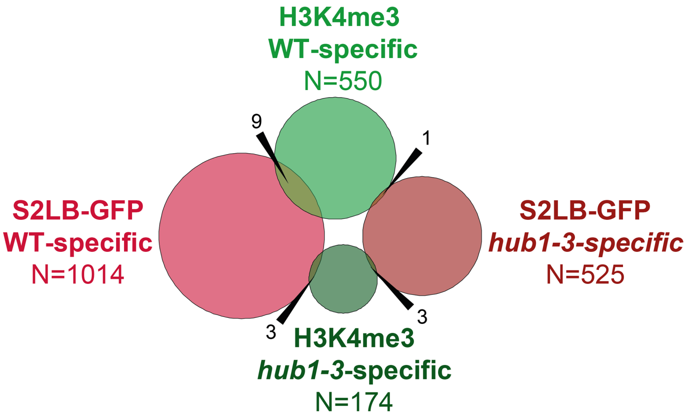

Figure S11. Detection of S2Lb-targeted genes losing a H3K4me3 peak in *hub1-3* seedlings. The Venn diagram shows the overlap between the lists of genes displaying specific marking by H3K4me3 or S2Lb in wild-type or in *hub1-3* seedlings. Only 9 genes appear to be subject to an H2B-H3 *trans*-histone crosstalk mediated by S2Lb, i.e. losing both H3K4me3 and S2Lb-GFP association in *hub1-3* plants. WT-specific: peaks identified in wild-type but not in *hub1-3* plants. *hub1-3*-specific: peaks identified in *hub1-3* but not in wild-type plants.

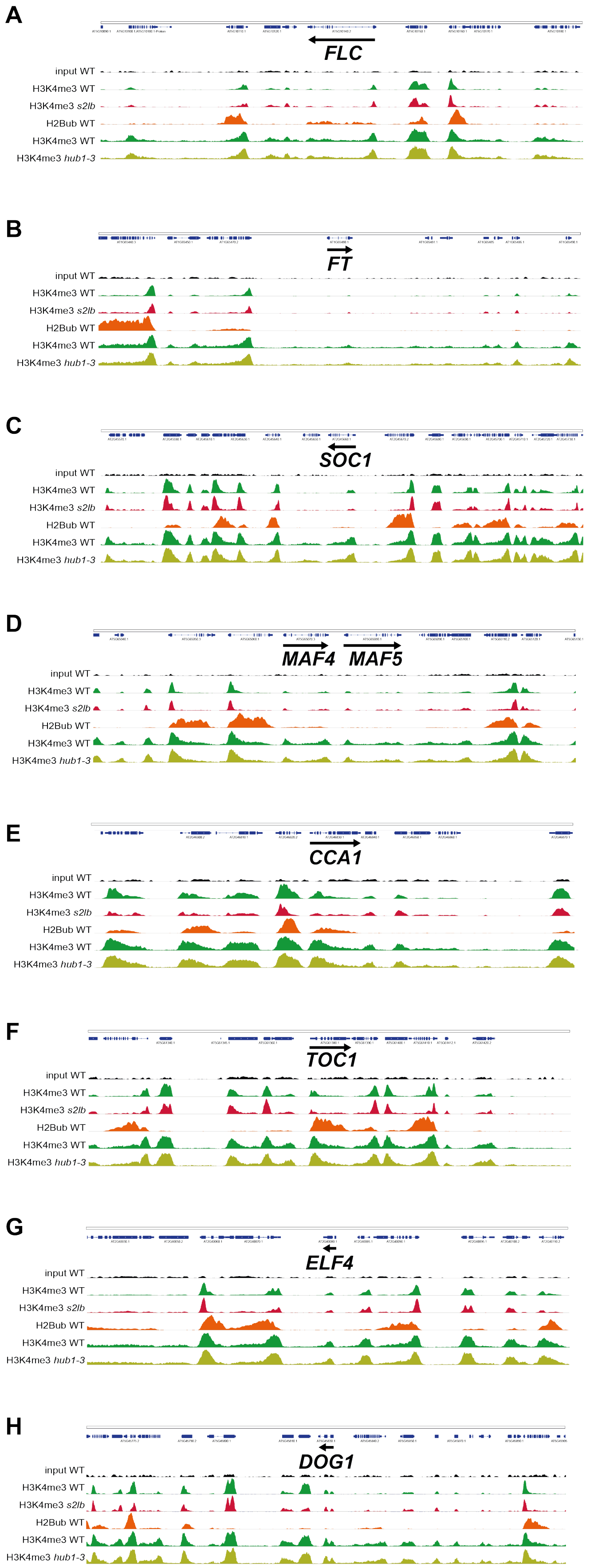

Figure S12. Genome browser snapshots of H3K4me3 and H2Bub profiles over representative genes in *s2lb-2* and *hub1-3* plants.
H3K4me3 signals in different genetic backgrounds are equally scaled.

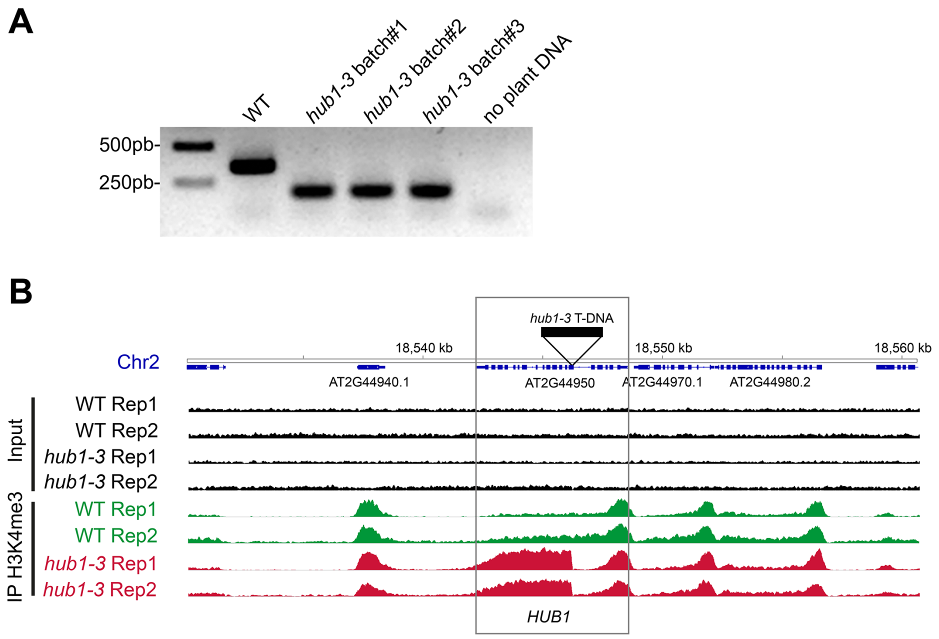

Figure S13. PCR genotyping and ChIP epigenotyping of the homozygous *hub1-3* allele in the seed batches used for ChIP-seq analyses in this study. A) PCR analysis of wild-type (WT) and *hub1-3* seed stocks used in H3K4me3 ChIP-seq experiments in Figure 6. DNA extraction and analysis were performed on a mix of 20 randomly picked seeds. **B)** H3K4me3 profile over the *HUB1* gene in the two independent biological replicates from the ChIP-seq analyses in Figure 6.
