# Supplementary material for: Arabidopsis S2Lb links AtCOMPASS-like and SDG2 activity in H3K4me3 independently from histone H2B monoubiquitination
Source: Genome Biol. 2019 May 21;20:100. doi: 10.1186/s13059-019-1705-4 (PMC6528313; doi:10.1186/s13059-019-1705-4)

Additional file 12. Original blots from Figure 3, 5, 6 and S5. All blots presented have not been cropped and the signals have not been modified. The same chromatin extract was loaded in parallel onto similar LiDs Tris-Tricine gels and blotted with the indicated antibodies.

Figure 3a

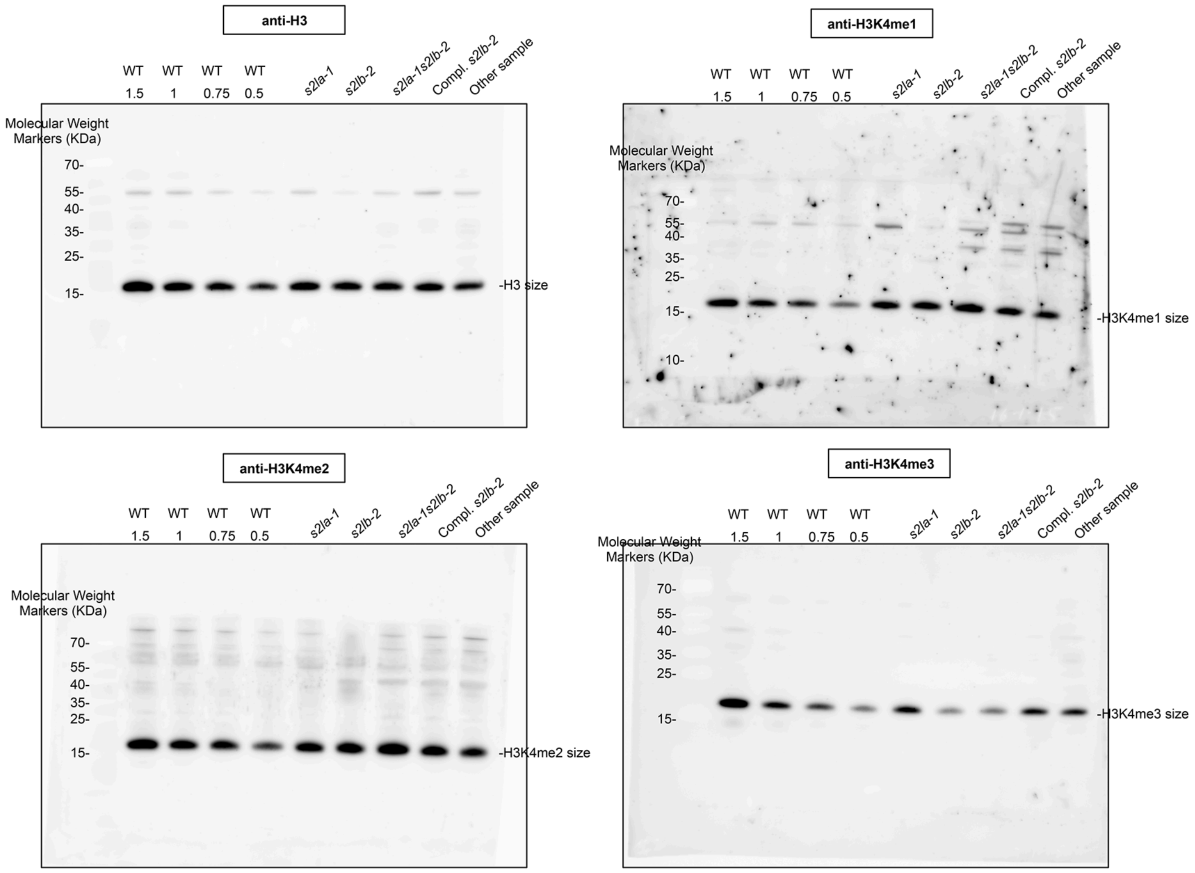

Figure 5a

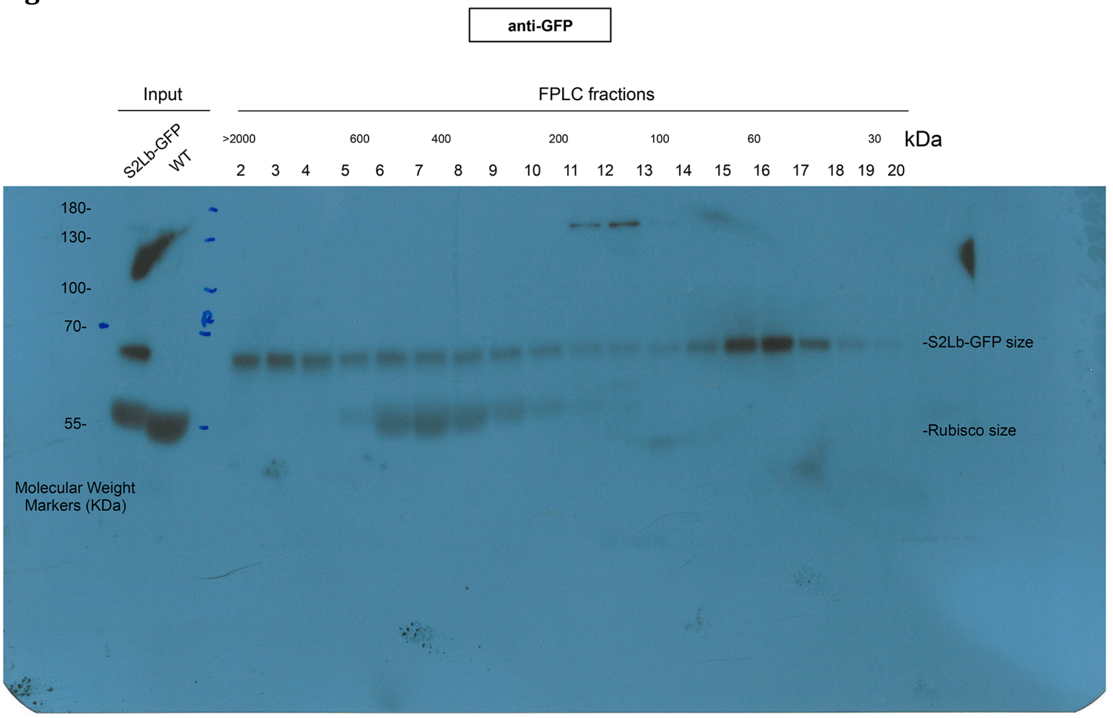

Figure 5b

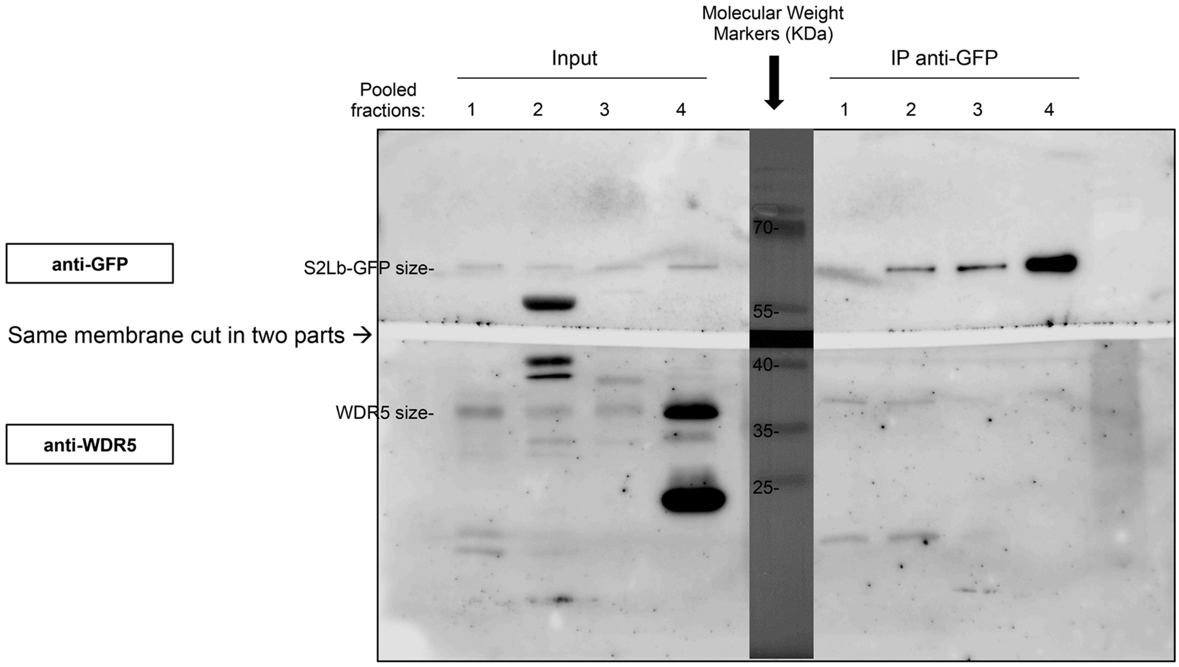

Figure 5d

A Long exposure

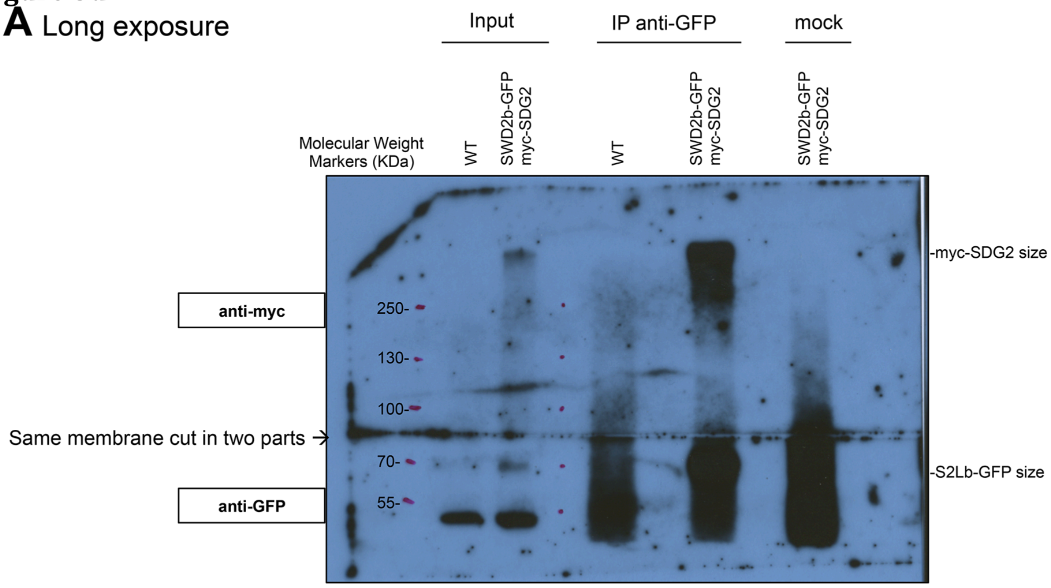

B Short exposure

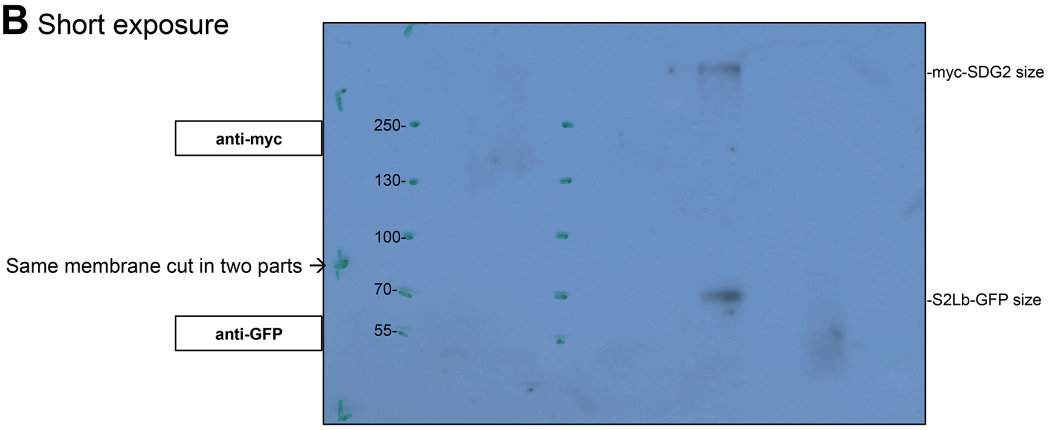

Figure 5e

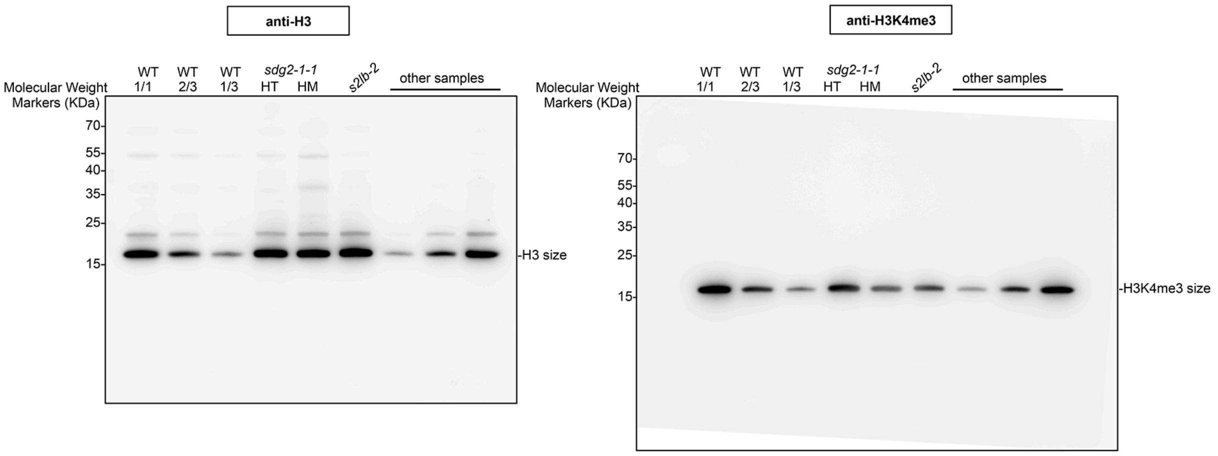

Figure 6b

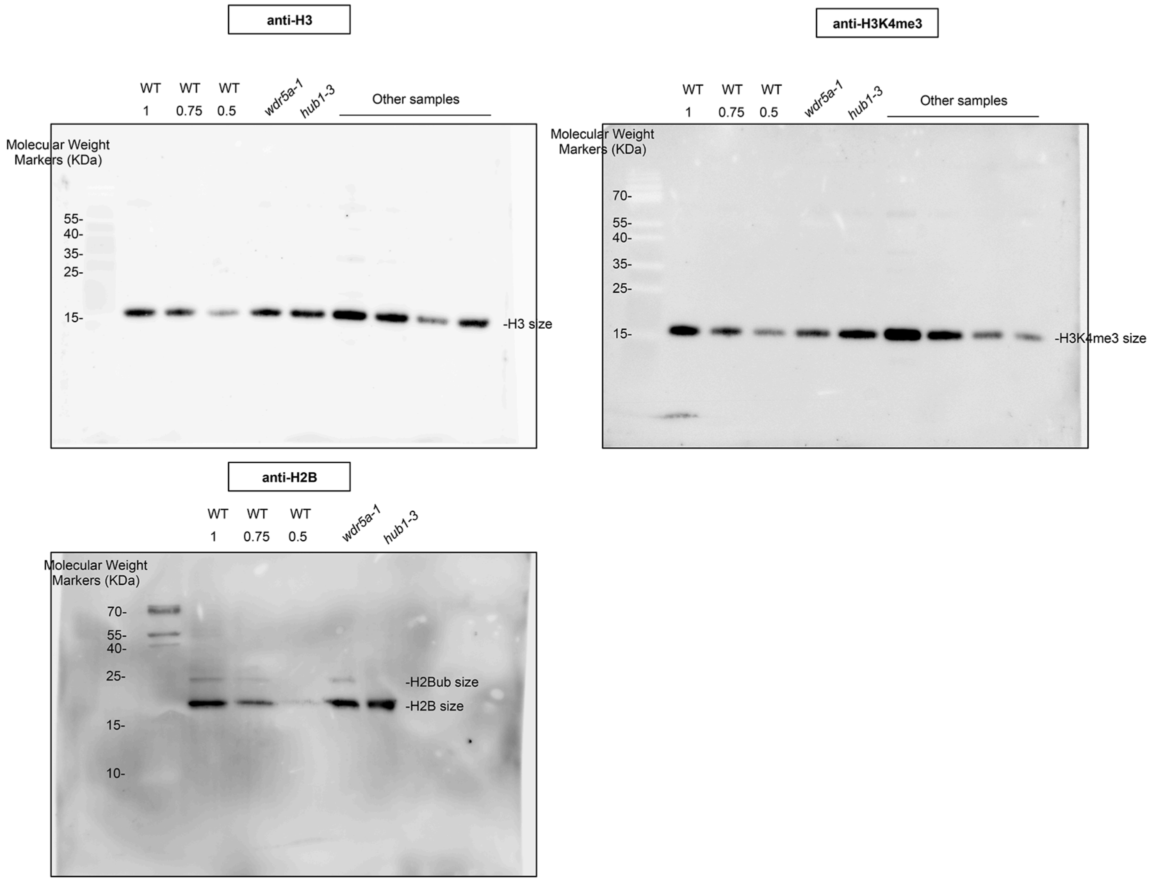

Figure 6c

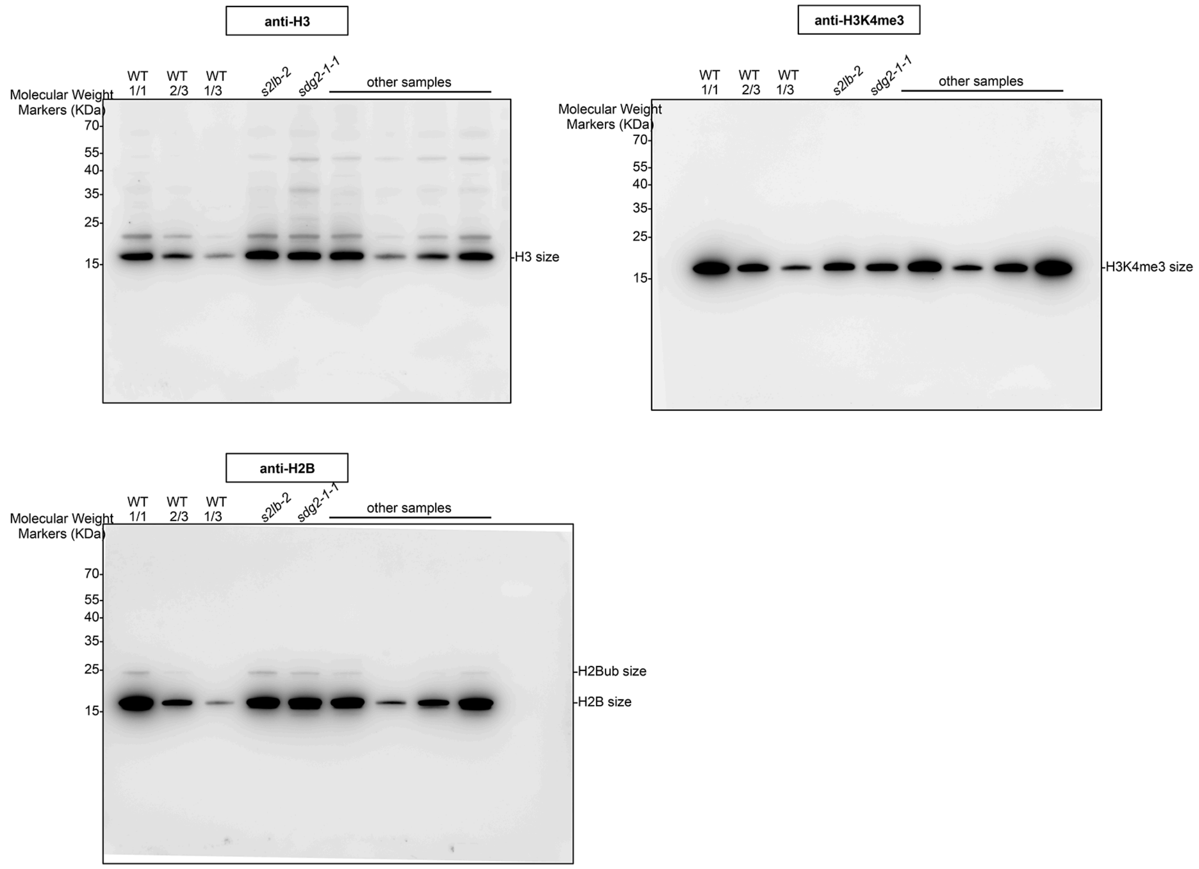

Figure S5

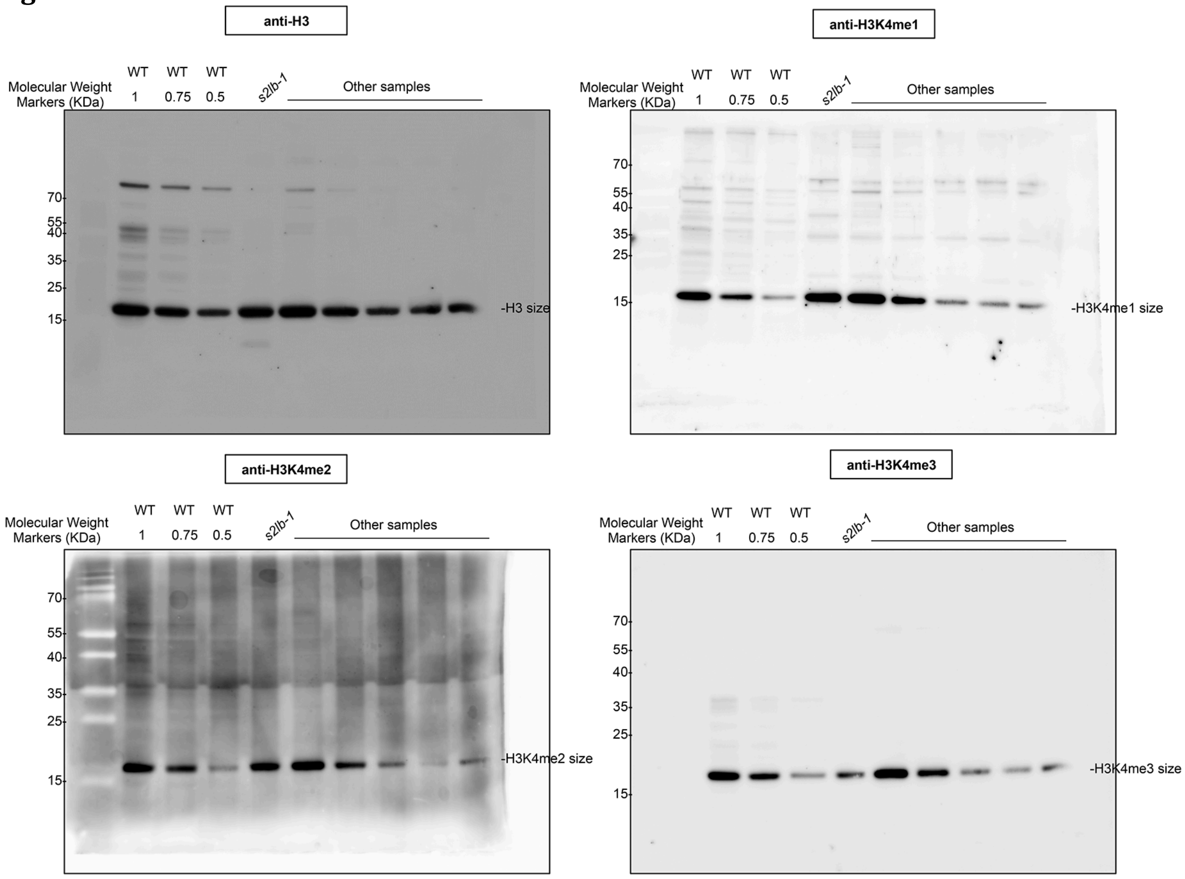

Supplement: Supplementary file 12 — Uncropped blots from Figure.3, 5, 6, and S5. (PDF 9903 kb) [file 13059_2019_1705_MOESM12_ESM.pdf]
