# Supplementary material for: Arabidopsis S2Lb links AtCOMPASS-like and SDG2 activity in H3K4me3 independently from histone H2B monoubiquitination
Source: Genome Biol. 2019 May 21;20:100. doi: 10.1186/s13059-019-1705-4 (PMC6528313; doi:10.1186/s13059-019-1705-4)
Supplement: Supplementary file 13 — Review history. (DOCX 57 kb) [file 13059_2019_1705_MOESM13_ESM.docx]

Review History

**First round of review**

**Reviewer 1**

**Are the methods appropriate to the aims of the study, are they well described, and are necessary controls included?**
Yes, they are well described and justified

**Are the conclusions adequately supported by the data shown?**

Yes.

**Are sufficient details provided to allow replication and comparison with related analyses that may have been performed?**

Genomic studies are described properly. The interaction experiments are also well described

**Does the work represent a significant advance over previously published studies? Please explain.**

As indicated in my report, this works has been carefully carried out and provides a significant advance in our understanding of the relationship between H3K4me3 and transcriptional regulation

**Is the paper of broad interest to others in the field, or of outstanding interest to a broad audience of biologists?**

Yes: The role of H3K4me3 in gene expression is of broad interest

**Comments to author:**

The H3K4me3 histone modification is one of the more abundantly found frequently associated with active transcription. The authors aim in this study at defining the possible mechanism of crosstalk of this modification with others (H2Bub) as well as to understand the role of SDG2. They identify genes that are coregulated between SDG2 and S2Lb, a member of the COMPASS complex, as demonstrated by the copurification of S2Lb with WDR5, one COMPASS subunit. They also analyzed the role of SDG2 and S2Lb in H3K4me3 using various combinations of genomic approaches and mutant plants. They conclude that H3K4me3 deposition occurs without the participation of an H2Bub-mediated pathway.

The experimental has been carefully carried out and most of the conclusions are sufficiently supported by the results obtained, although authors also leave some room for appropriate speculations in the Discussion section. While the text is generally easy to read, there are some sections are not, with jumps in the paragraphs that oblige the reader to go back and forth in the manuscript. I detect also some formal discrepancies, as specified below.

Comments

1.	Figure panels are named with capital letters in the text and lowercase in the images.

2.	Page 5, paragraph line 139. I think that the phenotypic differences between mutations in the S2La and S2Lb genes deserve further explanation/discussion (expression, redundancy…) if they have similar expression domains

3.	Page 7. Line 199. Change to "transcribed genes".

4.	Page 7, line 202. Define how the signal is considered "robust" (statistical analysis?).

5.	Page 7. Line 199, this section. Results show a link between S2Lb and H3K4me3, but is it exclusive? Please comment/discuss on the many locations of increased H3K4me3 in the absence of S2Lb binding.

6.	Page 7, line 224-. What favor selection of these genes by S2Lb? Is the presence of S2Lb cause or consequence?

7.	Page 8, line 237. I think I can understand the message but the sentence is obscure. Do the authors mean "highly transcribed in a few cells" in contrast to "transcribed in many cells", or else?

8.	Page 8, When starting to read this page I immediately expected to have an analysis of a few selected genes in detail, which was not the case until below, a couple of figure panels away. I think that rewriting of this part could be helpful.

9.	Page 8, line 253, Fig. 4F,G. The increase in H3K4me3 is apparent in the genes analyzed in Col-0 but not the mutants (4F). However, This does not seem to have a profound consequence on the transcription of these genes in the mutants (4G) where it is reduced by 30-40% only. Please explain/discuss.

10.	Page 8, line 262. Delete "majority", not sure that this is the case.

11.	Page 11, line 343. Again "robustness" needs some kind of statistical support.

**Reviewer 2**

**Comments to author:**

The manuscript by Fiorucci et al. describes a role of S2Lb for genome-wide H3K4 trimethylation in Arabidopsis. The authors found that S2Lb associates with several components of the H3K4 methyltransferase complexes known as COMPASS-like and functions to mediate H3K4me3 enrichments at thousands of loci, particularly those highly-expressed genes. In addition, the authors found that S2Lb-SDG2 mediated H3K4me3 deposition is independent of H2B monoubiqutination in Arabidopsis. These findings are interesting, but need additional results to substantiate them, as detailed below.

Major concerns:

1. In addition to being a component of COMPASS, SWD2 is also an integral subunit of a subcomplex of the Cleavage and Polyadenylation Factor (CPF) that is involved in 3' mRNA processing in yeast (Nedea et al. JBC, 278: 33000-10). It is well known that SWD2 plays dual roles in mRNA processing and H3K4 methylation in yeast (Dichtl et al. RNA 10:965-77; Cheng et al. MCB 24: 2932-43). Thus, it is very likely that the Arabidopsis SWD2 homologs, S2La and S2Lb, play dual roles in H3K4 methylation (as a component of COMAPASS like) and 3' mRNA processing (as a component of CPF). Additional experiments are required to determine whether S2Lb is a component of the Arabidopsis CPF and whether S2Lb is involved in 3' mRNA processing in Arabidopsis. The observed defects in the s2lb mutant in this study may be partly attributed to a defect in 3' mRNA processing, and some defects may not be related to COMPASS-mediated H3K4me3.

2. To solidify that S2Lb is a subunit of COMPASS-like with WDR5a, a ChIP-seq assay using S2Lb-GFP wdr5a-1 needs to be conducted to determine whether genome-wide occupancy of S2Lb-GFP is dependent on WDR5a/COMPASS-like.

3. Fig. 3a: the western-blot assays are not quantitative. Please quantify the blot signals. Otherwise, it is hard to see that there is a 2-fold reduction in s2lb-2 (vs WT), as the authors concluded.

4. Fig. 4f-g: the ChIP data in Fig. 4f needs a one-hour time point as all of the four genes examined were strongly induced within one hour (Fig. 4g).

5. S2Lb is required for DOG1 expression (Fig. 1j). Is S2Lb enriched at the DOG1 locus to mediate H3K4me3 for its expression control?

Minor points:

1. Fig. S4A: please include one more independent rescue line.

2. Please add a detailed description on EGS/formaldehyde double-crosslinking in the ChIP method section.

3. Line 380 needs a reference.

4. Page numbers are missing in Ref 66.

5. Fig. S11: it is difficult to understand this venn diagram; please revise it to improve reading.

**Authors Response**

**Point-by-point responses to the reviewers’ comments:**

Reviewer #1:

1. Figure panels are named with capital letters in the text and lowercase in the images.

*This has been corrected in all places.*

2. Page 5, paragraph line 139. I think that the phenotypic differences between mutations in the S2La and S2Lb genes deserve further explanation/discussion (expression, redundancy…) if they have similar expression domains.

*This point has been addressed in the last paragraph of the Discussion.*

*We now better highlight the observation that S2La and S2Lb genes have similar expression patterns, S2La being expressed much more weakly. Also answering to Reviewer 2 on a potential dual function of SL2b in COMPASS and CPF complexes, we now also discuss the hypothesis that the two paralogs might have been subjected to a functional specialization in plants as reported for S. pombe.*

3. Page 7. Line 199. Change to "transcribed genes".

*This has now been corrected.*

4. Page 7, line 202. Define how the signal is considered "robust" (statistical analysis?).

*This has been addressed as follows.*

*Peaks were first identified using MACS2. As detailed in the Methods this allowed detecting statistically significant enrichments as compared to the Input signal (qValue below 0.05 for S2Lb/SDG2). A few peaks were also found in the wildtype negative controls, which were subtracted from the peaks identified in each corresponding ChIP-seq experiment. Such false-positive peaks are not visible in the presented genome browser snapshots (Figure 4c) and also not visible on a genome-wide scale using our fully processed ChIP-seq datasets (accessible with the GEO accession number GSE124319).*

*For clarity, we now better described S2Lb/SDG2 peak detection in the Methods).*

*In addition, we introduced additional supplementary data in Figure S6 showing a so-called Fingerprint analysis of S2Lb and SDG2 enrichment onto chromatin. This allowed testing and comparing significant enrichments of these two factors in the different ChIP-seq datasets.*

*This additional information is further complemented by statistical and boxplot analyses comparing S2Lb-GFP and MYCSDG2 enrichment onto target genes between different ChIP-seq datasets in Figure S6.*

5. Page 7. Line 199, this section. Results show a link between S2Lb and H3K4me3, but is it exclusive? Please comment/discuss on the many locations of increased H3K4me3 in the absence of S2Lb binding.

*We concur with the observation that such a discrepancy is certainly meaningful.*

*ChIP-seq analyses identified that basically all S2Lb chromatin association perfectly matches H3K4me3 along the genome (97%; Figure 4a), but the reciprocal comparison is not true as the majority of H3K4me3 peaks do not overlap with a significant S2Lb peak (75% of them; Figure 4a). In the revised Results section, we modified the paragraph starting on line 230 to be more explicit on that discrepancy. At lines 370, we describe more clearly that "The reciprocal comparison is not true as the majority of H3K4me3 peaks do not overlap with S2Lb or SDG2 peaks."*

*Then, we tested the possibility that "S2Lb might only be detected on the most frequently transcribed genes among the various seedling cell types". Subsequent analyses using both RNA-seq and RNA Pol II ChIP-seq data indeed allow identifying a clear difference between H3K4me3 and S2Lb profiles: while H3K4me3 is found onto most transcribed genes, S2Lb is mainly found on highly-transcribed genes (Figure 4d). We further hypothesize that "S2Lb and SDG2 might only be detected over the genes most frequently transcribed among the various seedling cell types or formerly transcribed genes might retain H3K4me3 marking but not S2Lb". Addressing this second hypothesis likely represents a long task.*

6. Page 7, line 224-. What favor selection of these genes by S2Lb? Is the presence of S2Lb cause or consequence?

*This has been addressed as follows.*

*Obviously, a first potential guess described in the manuscript was the H2Bub mark itself. However, our ChIP-seq analyses showed no clear defect in S2Lb-GFP profiles in hub1-3 plants (Figure 6d). This is extensively discussed.*

*A second guess was a gene selectivity based on sequence context. We therefore performed extensive motif searches (MEME) to determine the most frequent sequences contexts residing at S2Lb. This identified a frequent link with GA stretches, which was also true for SDG2 peaks and did not depend on H2Bub. This has now been introduced in lines 308-310 and in a new Table S7.*

*We concluded that, as recently proposed for other COMPASS subunits, S2Lb and probably also SDG2, are targeted by transcription factors to specific loci in a spatio-temporal manner (Song et al., PNAS 2015) as mentioned in lines 434-438. If the TF mechanism holds true for S2Lb directly or in a COMPASS context, the corresponding motifs might rather be located at the transcription factors binding sites in the regulatory regions of target genes and not under S2Lb peaks.*

7. Page 8, line 237. I think I can understand the message but the sentence is obscure. Do the authors mean "highly transcribed in a few cells" in contrast to "transcribed in many cells", or else?

*We apologize for the lack of clarity.*

*The sentence in line 239 has been modified as follows: "We concluded from these observations that S2Lb prevalently occupies genes when they are highly transcribed".*

8. Page 8, When starting to read this page I immediately expected to have an analysis of a few selected genes in detail, which was not the case until below, a couple of figure panels away. I think that rewriting of this part could be helpful.

*This has been addressed as follows.*

*To address this concern and be more figurative on selected genes, we included the following sentence in the paragraph as well as the corresponding panels in Figure 4c: "For example, S2Lb-GFP profile perfectly matched H3K4me3 domains over house-keeping genes like TUBULIN8 but was not detected over non-expressed genes like FT (Figure 4c). Of note, similar*

*S2Lb-GFP and H3K4me3 peaks of low intensity were found at different locations along the dormancy gene DOG1, which likely result from its sense and antisense transcription start sites [57]".*

*In this way we also address a question of Reviewer 2 on the possibility that DOG1 is directly targeted by S2Lb.*

9. Page 8, line 253, Fig. 4F,G. The increase in H3K4me3 is apparent in the genes analyzed in Col-0 but not the mutants (4F). However, This does not seem to have a profound consequence on the transcription of these genes in the mutants (4G) where it is reduced by 30-40% only. Please explain/discuss.

*This has been addressed as follows.*

*We concur with the observation that such a discrepancy is counter-intuitive. This is discussed as the following in the manuscript: "Still, as for S2Lb, both HUB and PAF1c loss-of-function trigger weak transcriptomic defects in Arabidopsis.*

*Depletion of H3K4me3 has only marginal effects on gene expression in other species as well. Hence, H3K4me3 may contribute to the reinforcement of the active state of transcription and to the fine-tuning of genome expression during plant development and adaptive responses, …".*

*For the sake of clarity, we further introduced the following sentence in line 452 of the Discussion: "Investigating more precisely the effect of S2Lb or other COMPASS subunits on transcription efficiency would probably require a quantification of nascent transcripts production in a dynamic system such as de-etiolation or another cellular adaptive response".*

10. Page 8, line 262. Delete "majority", not sure that this is the case.

*This has been corrected.*

*Only the non-monomeric form is mainly present in the high molecular weight complex. The term "majority" has been deleted and the sentence has been rephrased to avoid any misunderstanding.*

11. Page 11, line 343. Again "robustness" needs some kind of statistical support.

*This has been addressed as follows.*

*We now provide additional supplementary data in Figure S6 showing statistical and boxplot analyses of H3K4me3 enrichment onto target genes and their comparison between different ChIP-seq datasets.*

Reviewer #2:

Major concerns:

1. In addition to being a component of COMPASS, SWD2 is also an integral subunit of a subcomplex of the Cleavage and Polyadenylation Factor (CPF) that is involved in 3' mRNA processing in yeast (Nedea et al. JBC, 278: 33000-10). It is well known that SWD2 plays dual roles in mRNA processing and H3K4 methylation in yeast (Dichtl et al. RNA 10:965-77; Cheng et al. MCB 24: 2932-43). Thus, it is very likely that the Arabidopsis SWD2 homolog S2La and S2Lb, play dual roles in H3K4 methylation (as a component of COMAPASS like) and 3' mRNA processing (as a component of CPF). Additional experiments are required to determine whether S2Lb is a component of the Arabidopsis CPF and whether S2Lb is involved in 3' mRNA processing in Arabidopsis. The observed defects in the s2lb mutant in this study may be partly attributed to a defect in 3' mRNA processing, and some defects may not be related to COMPASS-mediated H3K4me3.

*This is an important and relevant point.*

*We acknowledge the rationale for questioning the potential implication of mRNA processing defects in s2lb-2 phenotypes. Both biochemical and genetic evidence detailed below indicate that S2Lb is likely not a CPF component in Arabidopsis. We rather envisage this possibility for S2La. An example of functional specialization of two SWD2 paralogs in COMPASS or CPF has already been described for the yeast S. pombe (Roguev et al., Mol Cell Prot 2004), adding to the knowledge that CPF complex composition is variable among different eukaryotic groups as for example between yeast species and human (Millevoi et al., NAR 2010). Consequently, considering the editorial revision time frame we consider that investigating a hypothetical CPF activity of S2Lb, or more likely S2la, is out of the scope of our manuscript in which all functional and mechanistic investigations are focused on S2Lb. Still, in the manuscript we do not interpret s2lb-2 phenotypic defects as being directly linked to H3K4me3 deposition. We only describe these phenotypes and highlight their similarity with COMPASS and SDG2 effects. As detailed below we describe these concepts more clearly in the revised manuscript. We hope there is no potential misinterpretation any more.*

*The possibility of S2Lb dual function has first been assessed by undertaking an IP-mass spec analysis, aiming at identifying all possible S2Lb interacting proteins. Identifying SDG2 as the most abundantly co-purifying protein establishes a direct link with H3K4me3 deposition. In contrast, no CPF complex subunit was detected among the co-purifying proteins. Vice versa, TAP-tag purification of 6 different Arabidopsis CPF subunits by Zhao et al. (Plant Physiology 2009) did not identify S2La/b proteins. Also, while yeast SWD2 proteins mainly associate within a ~500kDa CPF complex and barely with the ~1000kDa SET1 (COMPASS) complex at room temperature (Dichtl et al., 2004), we found that S2Lb is mainly part of a ~1000kDa complex (Figure 5a). Genetic evidence also indicate that all predominant S2Lb mutant phenotypes are shared with COMPASS (WDR5a, ASH2R) and H3K4 trimethylation defective phenotypes (SDG2), most notably dwarfism, impaired fertility and early flowering. Moreover, sdg2-1 plants are sterile whereas all s2lb-1 and s2lb-2 seedlings are viable and display no visible phenotypic defects at early seedling stage. Hence, having milder phenotypic defects than SDG2 knock-out, it appears that S2Lb deficiencies can largely be explained by its function in H3K4me3 deposition. For this reason, we have not introduced the concept of a potential association of S2Lb with CPF in the manuscript.*

*In contrast, As reported earlier by Beris et al (2016), the most noticeable phenotypic defect of s2la-1 plants that we identified is a late flowering phenotype. This is also the case of Arabidopsis CPF mutants (Liu et al., Science 2010). In S2La mutant plants, this phenotype associates with increased transcript levels of the FLC flowering regulator, which might result from post-transcriptional defects on its antisense transcripts as observed in CPF mutant plants (Liu et al., Science 2010). These late flowering phenotypes are clearly distinct from SDG2 and S2Lb loss-of-function effects. Consequently, at this stage, we envisage a potential function of S2La in RNA processing. This possibility might be successfully addressed by Nanopore sequencing and GRO-seq approaches, as well as by IP-Mass Spec analyses of S2La interacting proteins. If successful, these efforts might produce interesting information in future studies. Hence, investigating a potential role of S2La in RNA processing is a very long task (minimum 6-12 months of work).*

*To address Reviewer 2 concern, we have introduced the following sentence in lines "In agreement with its role in H3K4me3 deposition, predominant phenotypes induced by S2Lb loss-of-function are shared with COMPASS [40, 43-45] and H3K4 trimethylation defective phenotypes (SDG2), most notably dwarfism, impaired fertility and early flowering [35, 36]. In contrast, s2la-1 plants are late flowering like CPF subunit mutants [82]. Hence, two SWD2 paralogs might be specialized in Arabidopsis, a situation previously identified in S. pombe [83]".*

2. To solidify that S2Lb is a subunit of COMPASS-like with WDR5a, a ChIP-seq assay using S2Lb-GFP wdr5a-1 needs to be conducted to determine whether genome-wide occupancy of S2Lb-GFP is dependent on WDR5a/COMPASS-like.

*We understand the rationale for this request as WDR5 might have a direct role in COMPASS chromatin association.*

*Nevertheless, addressing this mechanistic aspect requires crossing the WDR5a RNAi and the pS2Lb::S2Lb-GFP lines (about 3 months if successfully maintaining similar WDR5a knock-down efficiency), followed by ChIPs and NGS (minimum 2 months) and subsequent bioinformatics analyses. This is incompatible with the time frame for revision of the manuscript.*

*Authors are sincerely sorry for not being more positive on requests 1. and 2., but considering that our study already involves important efforts in genomic (10 different ChIP-seq plus replicates) and proteomic analyses, we sincerely believe that undertaking new ChIP-seq experiments to address this additional question goes beyond the scope of our manuscript. More generally, we do not see that question as an additional control of S2Lb association with COMPASS, which is already shown by protein-protein association studies and by comparative genomics of S2Lb, H3K4me4 and SDG2. As detailed below, all other issues from Reviewer 2 have been fully addressed.*

3. Fig. 3a: the western-blot assays are not quantitative. Please quantify the blot signals. Otherwise, it is hard to see that there is a 2-fold reduction in s2lb-2 (vs WT), as the authors concluded.

*This point has been addressed as follows.*

*We concur with the notion that western-blot assays are not as quantitative as presented in many studies. Still, we have used several precautions to obtain semi-quantitative results, including 1) image acquisition with digital luminescence imager, 2) capturing non-saturating signals and 3) performing a quantification of H3K4me3 signals relative to total histone H3 signals.*

*Furthermore, in Figure 3, a dilution series of the wild-type sample demonstrates that signals are visibly fit to the quantity of epitope loaded in the gel.*

*To address reviewer 2 request, we also present in Figure 3a the results of quantification of the H3K4me1, H3K4me2 and H3K4me3 signals relative to total histone H3 signals, which are calculated as the mean of 3 independent experiments. The whole results, including standard deviations, are also detailed in Figure S5a.*

4. Fig. 4f-g: the ChIP data in Fig. 4f needs a one-hour time point as all of the four genes examined were strongly induced within one hour (Fig. 4g).

*This point has been addressed as follows.*

*We concur with the observation that ChIP-qPCR assays at 6h might miss the best time point at which WDR5a and S2Lb effect on H3K4me3 is maximized. Consequently, we have performed a ChIP-qPCR analysis of the same genes after 1h of exposure to light. As observed previously (Bourbousse et al., PLoS Genet 2012), all the genes tested still had low, if any, H3K4me3 enrichment at this time point (see below in a figure for Reviewer 2). This could be interpreted by a few cells expressing these light-responsive genes at that early time point, preventing significant detection of H3K4me3 by ChIP.*

*Consequently, this result indicates that only the 6h time point -as used in Figure 4f (now Figure 4g)- allows to test for a significant defect in H3K4me3 enrichment in the COMPASS mutant lines.*

5. S2Lb is required for DOG1 expression (Fig. 1j). Is S2Lb enriched at the DOG1 locus to mediate H3K4me3 for its expression control?

*Yes, the DOG1 gene is targeted by S2Lb-GFP in our ChIP-seq experiments. A search of the gene ID (AT5G45830) in Table*

*S3 allows identifying DOG1 as an S2Lb-GFP target in both wild-type and hub1-3 backgrounds. To address more precisely the question by Reviewer 2 and a similar request on S2Lb association to specific genes by Reviewer 1, we now provide a snapshot of S2Lb-GFP profiles over the DOG1 locus in Figure 4c and in the revised Figure S12. Moreover, we described this observation at line 211: "Of note, similar S2Lb-GFP and H3K4me3 peaks of low intensity were found at different locations along the dormancy gene DOG1, which likely result from its sense and antisense transcription start sites [57]".*

Minor points:

1. Fig. S4A: please include one more independent rescue line.

*This point has been addressed as follows.*

*We now present the rosette phenotype of two independent complementation assays in which the s2lb-2 line is rescued either by a GFP-tagged or a native S2Lb protein. Of note, the s2lb/pS2Lb::S2Lb-GFP complemented line presented in Figure*

*S4 is the same one that we used for S2Lb-GFP ChIP-seq.*

*This information has been introduced in the revised Figure S4A and in the text on line 150.*

2. Please add a detailed description on EGS/formaldehyde double-crosslinking in the ChIP method section.

*This point has been addressed as follows.*

*A reference describing this method for Arabidopsis ChIP-seq is now given in the Methods section, as well as a detailed description of the two crosslinking steps.*

3. Line 380 needs a reference.

*This point has been corrected on line 391.*

4. Page numbers are missing in Ref 66.

*This has been corrected.*

5. Fig. S11: it is difficult to understand this venn diagram; please revise it to improve reading.

*This has been corrected.*

*The legends of the figure S11 were misleading and have been edited to better describe the Venn diagram meaning.*

**Second round of review**

**Reviewer 1**

The authors have addressed satisfactorily all my comments

**Reviewer 2**

My major criticisms (Points 1 & 2) have not been addressed satisfactorily. The authors argue that S2La and S2Lb may be functionally divergent, which is purely speculative. s2lb is from a Nossen background, and was subsequently introgressed into Col; hence, the phenotypic differences between s2lb-2 and s2la might be due to that a modifier (or modifiers) from Nossen may have been introduced into s2lb-2. I still think that a ChIP-seq assay using S2Lb-GFP wdr5a-1 needs to be conducted. Although the authors showed that WDR5a was co-purified with S2Lb, an analysis on the molecular function of this protein-protein association is required to prove that S2Lb indeed functions as part of COMPASS to regulate target gene expression.

**Authors Response**

**Point-by-point responses to the reviewers’ comments:**

Comment

Editor request: We do suggest that you address Referee 2’s comment on the different backgrounds when discussing possible divergent roles of S2la and S2lb.

*As requested, this concern has been carefully addressed by adding new sentences in the Discussion paragraph entitled "A diversity of COMPASS-like complexes in A. thaliana", in which we now clearly discuss the consequences of using different genetic backgrounds in our analyses.*

Editor request: Also we ask that you carefully check your claims with regards to Sl2b functioning as part of COMPASS and tone these down if necessary.

*This concern has been carefully addressed by checking all related mentions, and then by 1) modifying text in the Results paragraph entitled " S2Lb co-regulates a large set of genes with AtCOMPASS-like complexes and with SDG2", and 2) by modifying the Discussion paragraph entitled " S2Lb and COMPASS-like proteins as partners of the plant-specific histone methyltransferase SDG2".*
